# Efficacy and safety of Everolimus in children with TSC - associated epilepsy – Pilot data from an open single-center prospective study

**DOI:** 10.1186/s13023-016-0530-z

**Published:** 2016-11-03

**Authors:** Sharon Samueli, Klaus Abraham, Anastasia Dressler, Gudrun Gröppel, Angelika Mühlebner-Fahrngruber, Theresa Scholl, Gregor Kasprian, Franco Laccone, Martha Feucht

**Affiliations:** 1 Department of Pediatrics and Adolescent Medicine, Medical University of Vienna, Vienna, Austria; 2 Department of Biomedical Imaging and Image Guided Therapy, Medical University of Vienna, Vienna, Austria; 3 Institute of Medical Genetics, Medical University of Vienna, Vienna, Austria

**Keywords:** Tuberous sclerosis complex, Epilepsy, Child, mTOR, Everolimus

## Abstract

**Background:**

Epilepsy occurs in up to 90 % of all individuals with tuberous sclerosis complex (TSC). In 67 % disease onset is during childhood. In ≥ 50 % seizures are refractory to currently available treatment options.

The mTOR-Inhibitor Everolimus (Votubia®) was approved for the treatment of subependymal giant cell astrocytoma (SEGA) and renal angiomyolipoma (AML) in Europe in 2011. It’s anticonvulsive/antiepileptic properties are promising, but evidence is still limited. Study aim was to evaluate the efficacy and safety of Everolimus in children and adolescents with TSC-associated epilepsies.

**Methods:**

Inclusion-criteria of this investigator-initiated, single-center, open, prospective study were: 1) the ascertained diagnosis of TSC; 2) age ≤ 18 years; 3) treatment indication for Votubia® according to the European Commission guidelines; 4) drug-resistant TSC-associated epilepsy, 5) prospective continuous follow-up for at least 6 months after treatment initiation and 6) informed consent to participate.

Votubia® was orally administered once/day, starting with 4.5 mg/m^2^ and titrated to achieve blood trough concentrations between 5 and 15 ng/ml. Primary endpoint was the reduction in seizure frequency of ≥ 50 % compared to baseline.

**Results:**

Fifteen patients (nine male) with a median age of six (range; 1–18) years fulfilled the inclusion criteria. 26 % (4/15) had TSC1, 66 % (10/15) had TSC2 mutations. In one patient no mutation was found. Time of observation after treatment initiation was median 22 (range; 6–50) months.

At last observation, 80 % (12/15) of the patients were responders, 58 % of them (7/12) were seizure free. The overall reduction in seizure frequency was 60 % in focal seizures, 80 % in generalized tonic clonic seizures and 87 % in drop attacks.

The effect of Everolimus was seen already at low doses, early after treatment initiation.

Loss of efficacy over time was not observed.

Transient side effects were seen in 93 % (14/15) of the patients. In no case the drug had to be withdrawn.

**Conclusion:**

Everolimus seems to be an effective treatment option not only for SEGA and AML, but also for TSC-related epilepsies. Although there are potential serious side effects, treatment was tolerated well by the majority of patients, provided that patients are under close surveillance of epileptologists who are familiar with immunosuppressive agents.

## Background

Tuberous sclerosis complex (TSC) is a genetic autosomal dominant multi-system disorder, affecting 1–2 million people worldwide. TSC is characterized by benign tumor-like lesions in potentially all organ systems [1]. So far, TSC has been mapped to two genetic loci; TSC1 (located on chromosome 9q34, encoding for the protein hamartin) and TSC2 (located on chromosome16p13.3, encoding for the protein tuberin) [2, 3]. Hamartin and tuberin are widely expressed in all tissues, functioning as a tumor suppressor complex involved in the control of cell growth and division. The complex appears to interact with RHEB GTPase, thus sequestering it from activating mechanistic target of Rapamycin (mTOR) signaling, part of the growth factor (insulin) signaling pathway [4].

Pathogenic mutations in either of the two genes (TSC1 or TSC2) cause dysfunction of the intracellular hamartin/tuberin-complex, leading to over-activation of the mTOR signaling pathway resulting in uncontrolled protein synthesis and cell growth [4, 5].

The CNS is affected in more than 90 % of individuals with TSC, with the presence of characteristic lesions such as cortical or subcortical tubers, subependymal nodules (SEN), subependymal giant cell astrocytomas (SEGA), and white matter radial migration lines (RML) [6]. Neurological complications include obstructive hydrocephalus (due to SEGAs located near the foramen of Monroe), TSC-associated neuropsychiatric disorders (TAND) and epilepsy [7].

Epilepsy occurs in up to 90 % of all individuals with TSC. In 67 % disease onset is during childhood. According to the TSC management recommendations published in 2012 [8], treatment options for TSC-associated epilepsy in children and adolescents include various antiepileptic drugs (AEDs), with Vigabatrin being the drug of first choice, Adrenocorticotropic hormone (ACTH), epilepsy surgery, the ketogenic diet (KD) and vagus nerve stimulation (VNS) [9]. However, despite the growing number of recently licensed AEDs TSC-related epilepsies are still difficult to treat in over 50 % of cases [8–10]. In addition, only a small percentage of carefully selected TSC patients are ideal candidates for curative epilepsy surgery, and seizure freedom can only be achieved in about 56 % of them [11].

Growing knowledge about the molecular relationship between TSC and mTOR [12–15] led to the clinical testing of allosteric mTOR inhibitors. In 2011 Everolimus, a Rapamycin analogon, was approved as an orphan drug for the treatment of TSC patients with SEGAs and/or renal angiomyolipomas at risk for complications, but not amenable to surgery [16–18].

Clinical studies investigating the effect of mTOR inhibitors on TSC-related epilepsies are still limited and results are highly variable as both improvement and worsening of seizures have been reported [19–23]. In addition, there is clear evidence from animal and human treatment trials that the withdrawal of mTOR inhibitors leads to recrudescence of clinical symptoms, such as tumor regrowth or seizure recurrence [24–28]. Finally, mTOR inhibitors are associated with potentially serious adverse side effects that can compromise long-term tolerability and compliance [4]. Aim of the present study was the evaluation of both the efficacy and safety of Everolimus in children and adolescents with TSC - related epilepsies.

## Methods

Study inclusion criteria of this single center, open, prospective study were: 1) the ascertained diagnosis of TSC; 2) age ≤ 18 years; 3) treatment indication for Everolimus (Votubia®) according to the European Commission guidelines, (i.e. SEGA); 4) drug-resistant TSC-associated epilepsy, 5) a continuous prospective follow-up period of at least 6 months after initiation of Votubia®, and 6) informed consent to participate.

Seizure and syndrome classification was in line with the 2010 ILAE classification proposal [29].

Pharmaco-resistance was defined according to the ILAE consensus proposal [30].

Everolimus was orally administered once per day, starting with 4.5 mg/m^2^ and titrated to achieve blood trough concentrations (measured with the LC-MS/MS method) between 5 and 15 ng/ml.

Change of concomitant AEDs was not allowed during baseline and the first 6 months after initiation of Everolimus.

Follow-up visits were scheduled every 2 weeks during titration and at every 3 months during treatment. They included clinical internal, neurological and psychiatric examinations, seizure count (according to parents/caregivers seizure diaries) and EEG, as well as blood sampling.

Seizure frequency during the 3 months before initiation of Everolimus was defined as “baseline”.

Treatment response was defined as the median reduction in seizure frequency of ≥ 50 % at six, 12, 18 months and at last observation compared to baseline.

Side effects were evaluated using a structured questionnaire, covering the side effects reported during EXIST I and II [17, 18]. Side effects were graded I-V, according to the Common Terminology Criteria for Adverse Events (CTCAE), published by the National Cancer Institute [31].

MRI was performed according to the international guidelines [8] every 12 months and reviewed by an expert neuroradiologist (GK).

The study was reviewed and approved by the Medical University of Vienna ethics committee (ethics committee’s number: EK 1363/2014).

## Results

### Patient’s characteristics are shown in Table 1

**Table 1:** Patient’s characteristics

Patients	Gender	Mutation	CNS Manifestations	Age Epilepsy Onset	Duration of epilepsy prior to Everolimus	Antiepileptic drug therapy at baseline	VNS	KD	Prior Epilepsy Surgery	Prior SEGA Surgery	Age Everolimus Start
1	m	TSC1	SEGA, SEN, RML	4 years	2 years	TPM					6 years
2	m	TSC2	SEGA, SEN, RML	3 months	16 years 9 months	OXC					17 years 3 mths
3	f	TSC2	SEGA, SEN, RML	9 months	12 years 3 months	VPA, LEV, PHT					12 years 10 mths
4	f	TSC2	SEGA, SEN	6 months	5 years 6 months	LEV			y		6 years 2 mths
5	f	TSC2	SEGA, SEN	7 months	16 years 6 months	LEV, PGB	y				17 years 1 mths
6	m	TSC2	SEGA, SEN	3 months	6 years 9 months	RUF, ZNS	y				6 years 11 mths
7	f	TSC1	SEGA, SEN	2 years 9 months	16 years 3 months	OXC					18 years 6 mths
8	m	TSC2	SEGA, SEN	3 months	1 year 9 months	TPM, LEV, VGB					2 years 1 mths
9	m	TSC2	SEGA, SEN	2 years	4 years	VGB					6 years 8 mths
10	m	TSC1	SEGA, SEN, RML	4 months	14 years 9 months	OXC, PGB	y				15 years 6 mths
11	f	TSC1	SEGA, SEN, RML	2 years	2 years	OXC, VGB				y	3 years 11 mths
12	m	TSC2	SEGA, SEN	1 year 3 months	5 years	RUF	y				8 years 1 mths
13	m	TSC2	SEGA, SEN, RML	4 months	5 years 9 months	VPA					6 years 8 mths
14	m	TSC2	SEGA, SEN	4 months	0 year 9 months	VGB, LEV		y			1 year
15	f	negative	SEGA, SEN	1 year 3 months	2 years	RUF, TPM					3 years 3 mths

Seventeen patients were screened since April 2012; 1 patient had to be excluded due to compliance problems, another one did not fulfill the criteria for drug-resistant epilepsy [30].

Fifteen children and adolescents (9/15 male), with a median age of 6 (range; 1–18) years were finally included.

Twenty-six percent (4/15) had TSC1 and 66 % (10/15) had TSC2 mutations. In one patient no mutation was found.

All patients had SEGA and SEN, in 40 % (6/15) RML were present.

The median age at study inclusion was six (range; 1–18) years. 60 % (9/15) were ≤ 6 years old (median 6 years, range; 1–6) and 40 % (6/15) were > 6 years old (median 16 years, range; 8–18).

All patients had drug-resistant epilepsies, with a median seizure frequency of 30 (range: 1–410) seizures/month at baseline. Age at seizure onset was median 0.6 (range; 0.3–4) years. Disease duration before initiation of Everolimus was median 5.5 years (range; 0.75–16.75 years). The number of AEDs used before study inclusion was median 5 (range; 1–11). The median number of concomitant AEDs at baseline was 2 (range; 1–3). The AEDs used were Levetiracetam (5/15), Vigabatrin (4/15), Oxcarbazepine (4/15), Topiramate (3/15), Rufinamide (3/15), Phenytoin (1/15), Pregabalin (1/15) and Zonisamide (1/15). 4/15 had VNS and 1/15 patient had an additional KD. 1/15 had had incomplete SEGA surgery, and in 1/15 epilepsy surgery had been performed prior to the treatment with Everolimus.

The seizure types present at baseline were focal seizures in 67 % (10/15), generalized tonic clonic seizures in 47 % (7/15), drop attacks in 47 % (7/15) and atypical absences in 33 % (5/15). One patient had infantile spasms.

The epilepsy syndromes diagnosed at study inclusion were Lennox-Gastaut-Syndrome (LGS) in 47 % (7/15), focal epilepsies in 47 % (7/15) and West Syndrome in 1 patient.

Sixty-six percent (10/15) of the patients had a previous history of infantile spasms.

### Efficacy data are shown in Table 2

**Table 2:** Efficacy data; responders are marked with “*“

Patients Nr.	Seizures per months (median)					Duration of Everolimus Therapy (months)	Max. daily dose (mg/kg)	Median trough concentration (ng/ml)	Dosage (mg/m^2^)
	Baseline	6 months	12 months	18 months	Last Observation				
1*	30	21	21	50	15	22	7.5	5.9	9.8
2	30	16	42.5	-	20	17	10	4.9	4.6
3*	10	0	1	2	0	41	10	4.1	5.7
4*	30	0	0	0	0	33	5	5.6	5.9
5*	120	32.5	39.5	62.5	25	23	10	4.3	4.8
6*	410	0	0	0	32	50	7.5	2	5.8
7*	1	0	-	-	0	7	10	2.6	4.4
8	180	180	-	-	180	6	5	5.3	6
9*	16	10	2.5	1	0	41	5	8.4	4.7
10*	2	1.5	1	0	0	32	12.5	3.5	6.8
11*	30	0.5	-	-	0	14	4	1.5	2.6
12	90	90	-	-	50	10	10	3.2	8.4
13*	30	0.5	0.5	0	0	21	5	4.2	4.6
14*	149	211	62.5	46	63	23	4	7.7	7.2
15*	90	21.5	2	1.5	1	20	7.5	4.7	8.6

The observation period after initiation of Everolimus was median 22 months (range; 6–50 months). Follow-up after initiation of Everolimus was ≥ 6 months in all 15 patients, ≥ 12 months in 12 and ≥ 18 months in 10 patients.

After 6 months 53 % (8/15), after 12 months 83 % (10/12) and after 18 months 80 % (8/10) were responders. After 6 months 27 % (4/15), after 12 months 25 % (3/12) and after 18 months 40 % (4/10) were seizure free.

At last observation, 12/15 patients (80 %) were responders, 58 % of them (7/12) were seizure free.

The majority of our patients had daily seizures. The median number of seizure free days per 28-day period at baseline was 0 (range; 0–27). At 6 months the median number of seizure free days per 28-day period was 19.5 (range; 0–28), at 12 months 26 (range; 0–28 seizure free days per 28-day period), at 18 months 26.75 (range; 0–28 seizure free days per 28-day period) and at last observation 28 (range; 0–28 seizure free days per 28-day period).

In 20 % (3/15) of our patients (Table 2 patients 1, 2 and 14) we observed an increase in seizure frequency of 66,7 % (30 seizures/month at baseline vs. 50 seizures/month at 18 months), 41,7 % (30 seizures/month at baseline vs. 42,5 seizures/month at 12 months) and 41,6 % (149 seizures/month at baseline vs. 211 seizures/month at 6 months).

The maximum daily dose of Everolimus was 12.5 mg/kg per day, the median dose was 5.8 (range; 2.6–9.8) mg/m^2^ and the median trough concentration was 4.6 (range; 1.6–7.8) ng/ml.

Responders had a median trough concentration of 4.5 (range; 1.6–7.8) ng/ml, the median dose was 5.8 (range; 2.6–9.8) mg/m^2^. In non-responders the median trough concentration was 4.9 (range; 4.1–5.3) ng/ml, the median dose was 6 (range; 4.6–8.4) mg/m^2^.

The time to response was median 1 month (range: 0.5–2.5 months).

In responders the number of concomitant AEDs was reduced; median 1 (range: 0–2) at last observation compared with median 2 (range; 1–3) at baseline. In one patient, all AEDs were successfully withdrawn.

### Group differences

The overall percent reduction in seizure frequency was 60 % for focal seizures, 80 % for generalized tonic clonic seizures and 87 % for drop attacks. Atypical absences were not considered, because seizure counts were not reliable.

There was no significant effect of Everolimus on EEG in responders with focal seizures. Only in one patient (patient 11) the EEG became normal during treatment.

In patients with LGS the EEG showed no changes in 57 % (4/7) and in 43 % (3/7) the EEG improved from multifocal and generalized ED to focal ED.

There was only one infant (patient 14) with infantile spasms; Reduction in seizure frequency in this patient was 58 % (median seizure frequency: 149/month at baseline vs. 63/month at last observation). The EEG changed from hypsarhythmia to multifocal spikes.

The age group ≤ 6 years showed better response rates than the age group 7–18 years with median percent reductions in seizure frequency of 76.1 % (range; -41.6–100 %) at 6 months and 98.9 % (range; 0–100 %) at last observation compared to baseline. In contrast, the age group from 7 to 18 years showed median percent reductions in seizure frequency of 59.8 % (range; 0–100 %) at 6 months and 89.6 % (range; 33.3–100 %) at last observation.

There was no significant association between changes in SEGA volumes and seizure response.

We also did not find significant differences between responders and non-responders with respect to gender and mutation type (TSC1 versus TSC2). Further we did not find differences between responders and non-responders with respect to concomitant AEDs (i.e. inducers and non-inducers were equally distributed in both treatment groups).

### Safety

Grade I adverse events (AEs) were seen in 93 % (14/15) of the patients. The most commonly reported side effect was stomatitis, reported in 66 % (10/15). Fifty-three percent (8/15) of our patients developed dyslipidaemia (highest level: 295 mg/dl), hypertriglyceridemia (highest level: 326 mg/dl) appeared in 16 % (4/15) and leukopenia (lowest level: 2280 cells/μl) in 13 % (2/15). Dyslipidämia was transient in 50 % (4/8), hypertrigylceridemia in 75 % (3/4) and leukopenia in all cases (2/2). One patient had frequent viral infections (nasopharyngitis) during the winter.

Grade II AES (i.e. angina herpetica) occurred in 7 % (1/15).

Grade III AES were not observed.

Grade IV AES necessitating a temporary treatment stop were seen in 26 % (4/15) of the patients: Three patients had pneumonia, and in one patient extensive impetigo contagiosa occurred.

In summary, treatment with Everolimus was safe during the observation period of median 22 months (range: 6–50 months). Side effects were manageable by our team that is familiar with the drug and immunosuppressive therapies.

In three patients Everolimus was withdrawn; in two patients (patients 8 and 12) due to pending epilepsy surgery (after 6 respectively 10 months) and in one patient (patient 7) due to ongoing compliance problems (after 7 months). Increase in seizure frequency and/or severity after withdrawal of Everolimus was not observed.

## Discussion

Taking into consideration that epileptic seizures in patients with TSC are usually difficult to treat and that the children included into this study had been already refractory to various AEDs (median 5; range; 1–11) as well as other treatment options including epilepsy surgery, VNS and the KD, the response to Everolimus in this study was good to excellent.

In addition, the effect was stable throughout treatment, and loss of efficacy was not observed during observation periods of up to 50 months. Consequently, the concomitant AEDs were reduced from median two at baseline (range; 1–3) to median one at last observation (range; 0–3).

A number of case reports and retrospective case series as well as two prospective studies evaluating the effect of mTOR inhibitors in overall 162 patients with TSC-associated epilepsies so far showed variable results [17, 20, 21, 32–38].

The two largest trials included 145 patients. The effect on seizure frequency was a secondary endpoint (primary endpoint was the reduction in SEGA - growth), and significant limitations made the interpretation of the results difficult: In the EXIST-1 study no change in seizure frequency compared with baseline was observed [17], whereas Krueger et al. reported an increase in seizure free patients from 38.5 % (10/26) at baseline to 65.2 % (15/23) after 24 months’ treatment. At baseline 26.9 % (7/26) of the patients reported at least one seizure/day, after 24 months treatment this number decreased to 13 % (3/23) [32]. Cardamone et al. published a case series of seven patients and reported a reduction in seizure frequency of ≥ 90 % in one patient and of 50–90 % in four patients (57 %). The median duration of treatment in this study was 18 months [38].

Wiegand et al. described reductions in seizure frequency between 25 and 100 % in 4/7 cases. In one patient treatment had to be stopped after 1 month, because of exanthema. The median duration of treatment in this study was 20–36 months [37].

In a prospective, multicenter trial, Krueger et al also included 20 patients with TSC - related epilepsies (median age: 8 years; range; 2–21 years). The absolute duration of treatment was 12 weeks. 60 % (12/20) had a ≥ 50 % reduction in seizure frequency [20].

Our results in 15 patients treated with Everolimus up to 50 (median 22) months are comparable, in part better than those reported in the above mentioned studies.

Response to Everolimus appeared soon after initiation in most of our patients, the median time to response being one month.

Similar results were reported by others: Krueger et al described a statistically significant response only in the final maintenance period (4 to 8 weeks after treatment initiation) [20]. In their case report of a 9 year old girl, Perek-Polnik described a rapid and 100 % response within 6 weeks after the initiation of Everolimus [35]. This result may be of relevance when treating infants and children as prolonged duration of the active disease has significant irreversible developmental consequences.

In September 2016, the results of EXIST-3, a randomised, double-blind, placebo-controlled, multi-center study, investigating prospectively the effect and safety of Everolimus in 366 patients with TSC associated focal epilepsy, was published [23]. A reduction in seizure frequency of ≥ 50 % was observed in 24.8 % of the patients treated with median 5.2 mg/m^2^ (range; 1.3–14.5 mg/m^2^) and in 32.3 % treated with median 7.5 mg/m^2^ (range; 1.4–24.4 mg/m^2^).

The percentage of seizure-free patients was 5.1 % in the low-exposure and 3.8 % in the high-exposure group.

The results in our study were significantly better with a percent reduction in seizure frequency of 60 % in patients with focal seizures (70 % of them were seizure free at last observation). This difference could be due to the younger age of our patients, who were median 6 years old (range; 1–18 years, 60 % of them ≤ 6 years), whereas the median age of the patients included in EXIST-3 was 20.1 years (range; 2–56 years) and only 28 % of them were < 6 years of age.

The median duration of epilepsy in our study was 5.5 years (range; 0.75–16.75 years), which might had been shorter, than in the EXIST-3 patients. However, this data were not published [23].

According to the results of preclinical studies mTOR inhibitors might be less effective in reducing seizures once they started than in preventing seizures from ever developing as well as many of the pathological and molecular changes in the brain that likely promote epileptogenesis [39, 40]. Early treatment might therefore show even better results.

The effect of Everolimus on different seizure types varied in our patients. Focal seizures showed the lowest response rate, with an overall percent reduction in seizure frequency of 60 % compared with 80 % in generalized tonic clonic seizures and 87 % in drop attacks. However, this result has to be interpreted with caution because of the small number of patients investigated.

The effect of Everolimus did not seem to be dose dependent in our study. In the responder - group, the median dose was 5.8 (range; 2.6–9.8) mg/m^2^, whereas in the non-responder-group no additional effect was observed when doses were increased further. This is in contrast to the results obtained in the EXIST-3 study where the high-exposure group showed a better response; 32.3 % showed a > 50 % seizure reduction with a median dose of 7.5 (range; 1.4–24.4) mg/m^2^, versus 24.8 % in the low-dose group with a median dose of 5.2 (range; 1.3–14.5) mg/m^2^ [23].

The percentage of adverse events in our study was comparable to previous studies [17, 18, 23, 32]; Grade I adverse events occurred in 93 %, the most common reported AE was stomatitis. Grade IV AES, necessitating a temporary treatment stop, were seen in 26 % of the patients. In no case it had to be terminated. The effects on blood lipids and leukocyte counts were mild and transient.

Provided the therapy is managed by specialists, who are familiar with the drug and can handle the side effects, and a thorough education of the patients and caregivers, the treatment with Everolimus therefore seems to be safe and well tolerated.

### Limitations

Due to the small sample size only descriptive statistics was possible.

A further limitation was the inhomogeneity with respect to seizure types and epilepsy syndromes. In addition, all patients had severe long-lasting (median duration 5.5 years; range; 0.75–16.75 years) refractory epilepsies which might have biased the results.

## Conclusion

Many TSC patients suffer from drug-resistant epilepsy. Various pre-clinical and clinical studies demonstrated that loss of function mutations of the genes encoding for the natural mTOR inhibitors TSC1 and TSC2 lead to aberrant mTOR signaling with consecutive development of cortical malformations and epilepsy [19]. Preclinical studies demonstrated that treatment with mTOR inhibitors (e.g. Everolimus) has both anti-convulsive and anti-epileptogenic effects [41–43].

Taking into consideration its proven efficacy in other TSC-associated manifestations (primarily SEGA and AML), Everolimus might become a potent disease modifying compound targeting also TSC –associated epilepsy.

However, as most of the effects of mTOR inhibition cease after drug discontinuation, a life-long treatment might be necessary. Further multi-center phase III studies are therefore needed to confirm our results, as well as these of EXIST-3, and to evaluate the efficacy and long-term safety, including timing and duration of its administration and possible interactions with standard AEDs, especially in very young children with TSC associated West Syndrome as well as developmental aspects.

## Abbreviations

ACTH: Adrenocorticotropic hormone
AED: Anti - epileptic Drug
CBZ: Carbamazepine
CNS: Central nervous system
ECBZ: Eslicarbazepine
ED: Epileptic discharges
GK: Assoc. Prof. Priv.-Doz. Dr. Gregor Kasprian
GTCS: Generalised tonic clonic seizure
ILAE: International League against Epilepsy
KD: Ketogenic diet
LCS: Lacosamide
LEV: Levetiracetam
LGS: Lennox-Gastaud-Syndrome
mTOR: Mechanistic target of Rapamycin (formally called: mammalian target of Rapamycin)
OXC: Oxcarbazepine
PER: Perampanel
PGB: Pregabalin
PHT: Phenytoin
RML: Radial migration lines
RUF: Rufinamide
SEGA: Subependymal giant cell astrozytoma
SEN: Subependymal nodule
TPM: Topiramate
TSC: Tuberous sclerosis complex
VGB: Vigabatrin
VNS: Vagus nerve stimulation
VPA: Valproic acid
